# A Comparative Study of Two Folate-Conjugated Gold Nanoparticles for Cancer Nanotechnology Applications

**DOI:** 10.3390/cancers2041911

**Published:** 2010-11-18

**Authors:** G. Ali Mansoori, Kenneth S. Brandenburg, Ali Shakeri-Zadeh

**Affiliations:** 1 Department of Bioengineering, University of Illinois at Chicago, 851 S. Morgan St. (MC 063), Chicago, IL 60607, USA; E-Mail: kbrand@uic.edu; 2 Department of Medical Physics, Tehran University of Medical Sciences, Tehran, Iran; E-Mail: shakeri2005@gmail.com

**Keywords:** 4-aminothiophenol, 6-mercapto-1-hexanol, cancer nanotechnology, folate, folate receptor, folic acid, gold nanoparticle, nanoconjugate, photothermal treatment

## Abstract

We report a comparative study of synthesis, characteristics and *in vitro* tests of two folate-conjugated gold nanoparticles (AuNP) differing in linkers and AuNP sizes for selective targeting of folate-receptor positive cancerous cells. The linkers chosen were 4-aminothiophenol (*4Atp*) and 6-mercapto-1-hexanol (*MH*) with nanoconjugate products named *Folate*-*4Atp*-*AuNP* and *Folate*-*MH*-*AuNP*. We report the folate-receptor tissue distribution and its endocytosis for targeted nanotechnology. Comparison of the two nanoconjugates’ syntheses and characterization is also reported, including materials and methods of synthesis, UV-visible absorption spectroscopic measurements, Fourier Transform Infra Red (FTIR) measurements, Transmission electron microscopy (TEM) images and size distributions, X-ray diffraction data, elemental analyses and chemical stability comparison. In addition to the analytical characterization of the nanoconjugates, the cell lethality was measured in HeLa (high level of folate receptor expression) and MCF-7 (low level of folate receptor expression) cells. The nanoconjugates themselves, as well as the intense pulsed light (IPL) were not harmful to cell viability. However, upon stimulation of the folate targeted nanoconjugates with the IPL, ~98% cell killing was found in HeLa cells and only ~9% in MCF-7 cells after four hours incubation with the nanoconjugate. This demonstrates that folate targeting is effective in selecting for specific cell populations. Considering the various comparisons made, we conclude that *Folate*-*4Atp*-*AuNP* is superior to *Folate*-*MH*-*AuNP* for cancer therapy.

## 1. Background and Introduction

A need exists to target cancer treatments specifically to the tumor site, without damaging healthy tissue. The answer to solving this challenge lies in the successful application of nanotechnology to cancer treatment [1]. Nanotechnology is based on the 1–100 nanometer scale as its name implies [2,3]. Since nanotechnology exists well below the size of the cell (10,000–100,000 nm), it is a great candidate for solving such problems. Due to its size, nanotechnology offers the potential to selectively seek out and destroy cancerous tissues by a variety of targeting and destruction methods.

A malignant cancer is typically fast growing, which means that it requires more nutrients and increased waste removal than healthy tissues. Nanoparticles injected into the bloodstream can enter into tumors because of the defects or pores within the tumor vasculature. However, the residence time within the tumor is limited due to random diffusion into and out of the tumor. Therefore, increasing the residence time of nanotechnology-based cancer treatments within tumors is necessary. To achieve increased residence time, active targeting must be used. Active targeting is based on selectively targeting cancer cells through a specific binding site on the surface of the cell, such as a receptor. Various methods of targeting cancer cells have been proposed [1,4]. One promising targeting method is with folic acid or folate, the folic acid salt, which is the subject of the present report.

**Figure 1:**
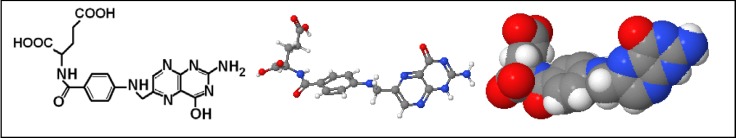
The molecular structure of folic acid.

Folic acid is also named pteroylglutamic acid and has the closed chemical formula C_19_H_19_N_7_O_6_ (Mw = 441.4 Da) and open chemical structure as shown in Figure 1.

Folic acid or folate (pteroylglutamate) is water-soluble and is brought into both healthy and cancerous cells by the folate-receptor. This receptor is used to transport folate into the cytosol for the synthesis of thymine by dihydrofolate reductase. The presence of the folate-receptor on a cell’s surface is regulated by the cell’s function. Cancer cells tend to overexpress the folate-receptor because of their vast requirement for folate. It has been proposed that the folate-receptor makes for a suitable targeting agent because of its relatively low expression level in healthy tissues and overexpression in cancerous tissues.

A positive aspect of folate is its possible conjugation with a number of nanotechnology platforms, such as gold nanoparticles and chemotherapeutic agents. When these nanotechnology platforms are deposited at the tumor site a variety of methods to eradicate the cancer cells can be used, such as thermal ablation, drug release or delivery, or even coating the cancer cells with a high affinity antigen, which the body’s immune system can detect and mount a defense against. Due to folate’s promising characteristics of non-immunogenic, specificity for cancer, and its possible conjugation with gold nanoparticles (AuNP) as reported here, folate-AuNP nanoconjugate is a front-runner as a targeting moiety for many cancer nanotechnology treatments.

### 1.1. Folate-Receptor Tissue Distribution

The folate-receptor is a glycosyl-phosphatidylinositol linked membrane protein with a molecular weight of 38,000 Daltons. Immunocytological tests have shown the presence of folate-receptor in the ovaries, kidneys, lungs, thyroid, the fallopian tube, as well as several ovarian cancers. According to Weitman and Kamen [5], the folate-receptor is commonly expressed within several healthy tissues of the body. Among the most positive tissues that express the folate-receptor are the choroid plexus, kidney, and the lungs. If these healthy tissues commonly express the folate-receptor then how would a cancer treatment targeting the folate-receptor be effective? The answer lies in the biology of the vasculature of healthy and cancerous tissue as well as the membrane localization of the folate-receptor. The most important part of this answer is the membrane localization. Epithelial cells, those forming a layer separating the body’s tissues from either the outside (skin) or from the interior cavities such as the gastrointestinal tract or lungs, have two distinct membrane forms. One (the basal membrane) is facing the other tissues or the bloodstream and the other (apical membrane or luminal surface) is facing the outside or cavity within the body. The folate-receptors within healthy tissues are localized on the apical membrane of the epithelial cells. This means that the folate-receptors in the choroid plexus are entirely expressed toward the cerebrospinal fluid. Likewise, for the kidney and lungs the folate-receptors are expressed toward the urine and air, respectively [6]. This was also found to be true in the gastrointestinal tract.

The secondary part of the answer to the original question is in regard to the vasculature differences between the healthy and cancerous tissues. As mentioned above, the vasculature of tumors is filled with defects, which are in the hundreds of nm size range, whereas the pores in the healthy tissue vasculature are in the few nanometer size range. This means that any targeted nanotechnology treatment agent must be larger than 10 nm to prevent the particle from entering healthy tissue. Also, the treatment agent must be smaller than 100 nm to allow it to enter the tumor. Therefore, the size of the nanoparticle treatment agent should prevent it from accessing the folate-receptor in healthy tissue. This may not apply if the patient has tissue damage, where the vasculature is damaged, or has been exposed to permeability enhancers, such that pore size is increased in healthy tissues. Since the nanoparticle treatment agent will be able to diffuse into the tumor through the defects in the tumor vasculature, the particles will only be exposed to the folate-receptor on the cancer cells [7].

It was shown that certain cancer cells overexpress the folate-receptor on an order of 100-times more than normal healthy cells [8]. This overexpression of folate-receptor in the cancer cells makes folate a suitable targeting agent for such cancer cells. It should be noted that malignant cancer, which continues growing into the surrounding tissues, would not only express a high number of folate receptors on its surface but also has high folate-receptor. The limited and well localized tissue distribution of the folate-receptor within healthy tissues, the defects in tumor vasculature, and the high expression level within several types of cancer make folate an appropriate choice of targeting moiety for nanotechnology based cancer treatment.

### 1.2. Folate-Receptor Endocytosis for Targeted Nanotechnology

Understanding the molecular mechanisms of the folate-receptor endocytosis within tumors allows for the selective targeting of cancer cells [8]. In humans, the normal blood concentration of folate is approximately 6.8–36 (nanomol/L (3–16 nanogram/mL) and is regulated by the kidney [6]. It has been estimated that 400 µg of folate must be replaced by dietary intake every day [9]. When folate is consumed the blood concentration will normally increase dramatically, and will almost immediately begin to be filtered out by the kidneys. Before the kidneys filter the excess folate, the cells of the body can uptake it if folate can bind to the cells’ folate-receptors.

Folate has a strong binding affinity for its receptor. Folate has an association constant of K_A_ = 2 × 10^7^ [*s*], *i.e.*,

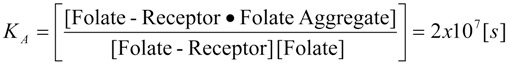
(1)
as reported by Hong *et al*. [10]. The association constant describes the bonding affinity between folate and its receptor at equilibrium. The binding and association of folate is important when designing the nanoparticle construct that targets the folate-receptor.

**Figure 2:**
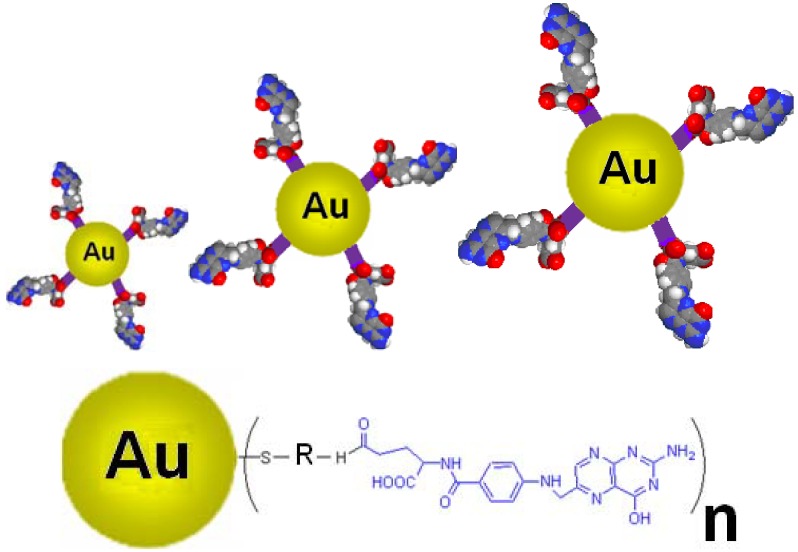
Schematics of a nanoconjugate of gold nanoparticle with folate (AuNP-Linker-Folate).

We know the vasculature within tumors contain many defects, which allow particle sizes around and below 100 nm to be passively deposited at tumor sites [11,12,13,14]. Passive targeting allows for increased depositing of nano-carriers within the tumor but does not guarantee their cellular uptake [15]. When the nano-carrier is passively targeted to tumors, it can remain within the tumor or it can also diffuse out of the tumor and back into the bloodstream, due to the high interstitial pressure within solid tumors and random diffusion [16]. Therefore, in order for the nano-carriers to achieve a greater affinity to, and residency time in, the tumor, as well as enter the cancer cells themselves, active targeting must be employed. By utilizing the cancer cells own deficiencies, need for folate, against themselves various nanoparticles can be used to target cancer cells [1,4,17,18,19,20]. In this project we choose the gold nanoparticle due to its unique properties. Folate can be conjugated to gold nanoparticles (nanoconjugate) through a linker as shown in Figure 2.

**Figure 3:**
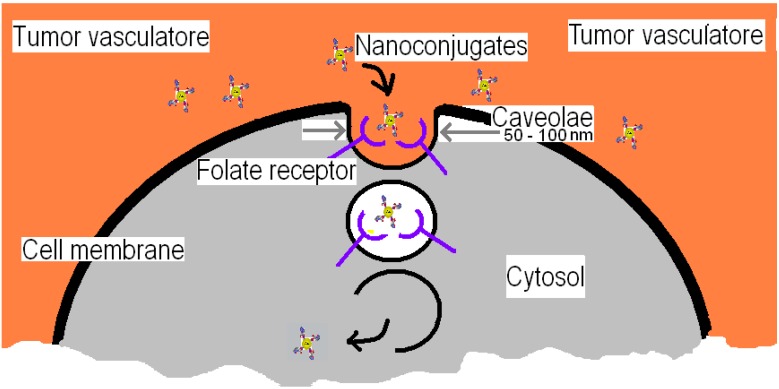
Various stages of (AuNP-Linker-Folate) nanoconjugate transfer in the cell through the folate-receptor on the cancer cell’s membrane.

The nanoconjugate is internalized by the folate-receptor (see Figure 3) in an endocytotic pathway, which does not enter into a clathrin coated pit pathway. This means that the nanoconjugate is released directly into the cytosol rather than being transported to an endosome or lysosome by intracellular vesicle transport.

The folate-receptor is linked in the lipid region of the membrane allowing it the ability to migrate through the membrane to release its content into the cytosol. Each caveolae is estimated as having approximately 750 folate-receptors in it, giving an average receptor density of 32,000/μm^2^ on each cancer cell. Figure 3 shows the caveolae beginning to close after the folate binds to the folate-receptor. The closed caveolae will begin to migrate to the interior surface of the phospholipid bi-layer. During this migration, the interior of the caveolae becomes more acidic reaching a pH of approximately 5, which causes dissociation of the *(AuNP-Linker-Folate)* nanoconjugate from the folate-receptor. The nanoconjugate is then released into the cytosol of the cancer cell upon reaching the interior surface of the cell membrane. Once the nanoparticles are internalized by the cancer cells, the cytotoxic agents can be released or in the case of gold nanoparticles, thermally ablate the cancer cells.

Important aspects to notice in this endocytotic pathway are the acidification of the interior of the caveolae during migration, the size of the caveolae, the absence of forming a vesicle for intracellular transport, and the dumping of the nanoconjugate directly into the cytosol. All of these features can be utilized in the final design scheme of a nanotechnology-based treatment of cancer.

Due to its possibility of conjugation, non-immunogenic properties, and requirement for cancer cell growth, folate is a novel targeting agent for malignant tumors. For the *in vivo* stage of our proposed work we may use different shaped and sized AuNPs including the advanced Au nanorods [21] and Au nanoshells [22]. *AuNPs* heat up as a result of absorption of visible light, which makes them suitable for use in cancer photothermal treatment.

### 1.3. Nanoconjugates Synthesis and Characterization

We developed and reported the original bioengineering design of this cancer nanotechnology process in 2005 and we reported our results in early 2006 [23]. Simultaneously, we developed a biosynthesis method for large scale production of metallic nanoparticles [24]. As a result of our research we have completed six research projects [25,26,27,28,29,30]. Meanwhile, two related papers by other groups [20,21] have reported on other folate-AuNP nanoconjugates. Here we report and compare two different folate-AuNP nanoconjugates that we have produced and tested. This includes comparison of their synthesis, characteristics and *in vitro* tests. The two nanoconjugates are named *Folate*-*4Atp-AuNP* and *Folate*-*MH-AuNP*, which are the results of conjugation of *AuNP* with folate by 4-aminothiophenol (*4Atp*) and 6-mercapto-1-hexanol (MH), respectively.

## 2. Materials and Methods

The reagent grade chemical and biological compounds that are used in this research are as follows: Hydrogen tetrachloroaurate (III) trihydrate (*HAuCl_4_3H_2_O*), 4-aminothiophenol (*C_6_H_7_NS*), 6-mercapto-1-hexanol (*C_6_H_14_OS*), Sodium borohydride (*NaBH_4_*), N, N'-dicyclohexylcarbodiimide (*C_13_H_22_N_2_*), Folic acid (*C_19_H_19_N_7_O_6_*), Trypan blue, RPMI 1640, MTT (3-[4,5-dimethylthiazol-2-yl]-2,5-diphenyltetrazoliumbromide), dimethylsulfoxide (*DMSO*), streptomycin, penicillin and trypsin-EDTA, Fetal calf serum (FCS), and HeLa and MCF7 cell lines.

**Figure 4:**
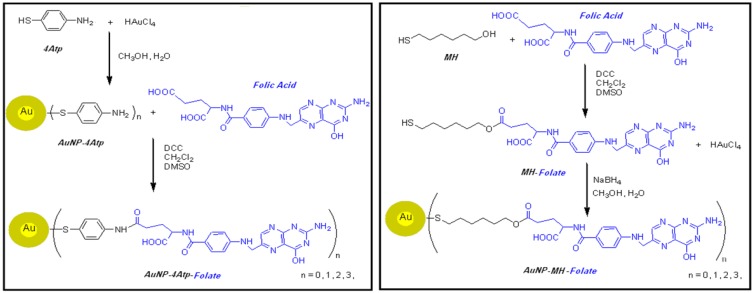
Schemes for the synthetic procedures of folate conjugated with AuNP using 4Atp (left scheme) [25,26,27] and MH (right scheme) [28,29] as the linkers to produce nanocongugates.

The details of the reagents’ commercial makers, cell culture procedure, synthesis and preparation of Folate-4Atp-AuNP and Folate-MH-AuNP nanoconjugates are already reported [25,26,27,28,29,30]. In Figure 4, schemes for the synthetic procedures of the two nanocongugates are shown on a comparative basis.

The final products of the synthesis are Folate-4Atp-AuNP nanoconjugate in powder form, exhibiting a golden color in reflection, and Folate-MH-AuNP nanoconjugate, also in powder form, with a deep brown color. The synthesis powder results were stored for further characterization and *in vitro* tests as described below.

### 2.1. Characterization of Nanoconjugates

For characterization of nanoconjugates, the following tests were performed: (i) UV-visible (UV-vis) absorption spectroscopy; (ii) Fourier Transform Infra Red (FTIR) measurements; (iii) Transmission electron microscopy (TEM); (iv) X-ray diffraction (XRD) and; (v) Elemental analyses. Details of these techniques and analysis were reported earlier [25,26,27,28,29,30]. The comparative results of the two nanoconjugates are reported blow.

#### 2.1.1. UV-Vis Spectroscopy

The UV-visible absorption spectroscopic measurements were recorded on a single beam UV-vis spectrometer, Agilent 8453, using quartz cells of 1 cm path length and methanol as the reference solvent at room temperature. It is known that *AuNP*s possess the characteristic surface plasmon absorption at 520 nm in the UV-visible absorption spectrum. This characteristic absorption band in the assemblies of *AuNP*s interlinked by various molecules may shift exhibiting a peak between 520 and 620 nm. Because of the propensity for intermolecular hydrogen bonding in the assemblies of *AuNP*s interlinked by different ligands, resultant broadening and red-shifting of the plasmon absorption peak are to be expected [24,31,32].

**Figure 5:**
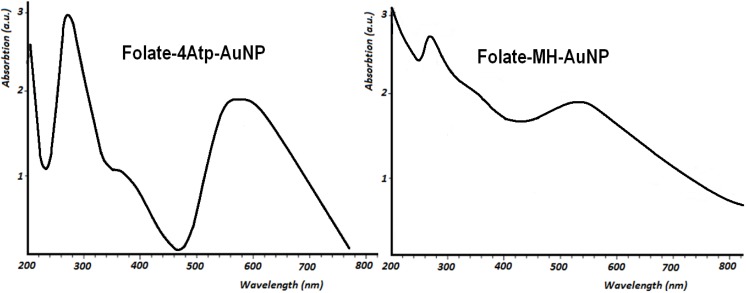
UV-Visible absorption spectra of Folate-4Atp-AuNP [25,26,27] and Folate-MH-AuNP [28,29] nanoconjugates.

Figure 5 shows the UV-vis spectra of *Folate-4Atp-AuNP* and *Folate-MH-AuNP* nanoconjugates. In the spectra of both nanoconjugates, the absorption maxima at 280 and the saddle points at 360 nm are confirmations of the covalent attachment of the folate with A*tp*-*AuNP* and *MH*-*AuNP* [33]*.* For the photothermal ablation of cancer cells reported below, a Lumenis intense pulsed light (IPL) source was used. Since the absorption peaks at ~560 nm in both nanoconjugates pertain to *AuNP* then the 560 nm filter of IPL source for irradiating of samples were found appropriate. According to Figure 5, the absorption peak of *Folate-4Atp-AuNP* is much sharper than that of *Folate-MH-AuNP*, but the level of absorption for both at ~560 nm are identical.

#### 2.1.2. Fourier Transform Infra Red Spectroscopy

**Figure 6:**
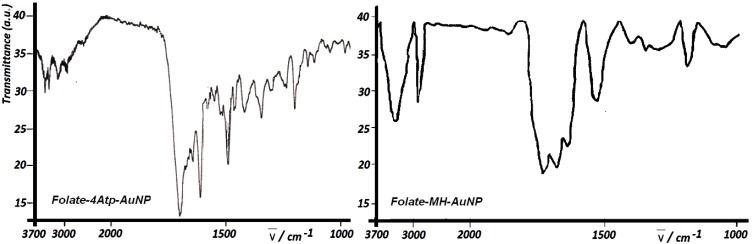
Fourier Transform Infra Red (FTIR) spectra of Folate-4Atp-AuNP [25,26,27] and Folate-MH-AuNP [28,29].

The Fourier Transform Infra Red measurements of the two nanoconjugates were recorded on a Shimadzu FT-IR 4300 instrument using KBr pellets at room temperature. Figure 6 shows the FTIR spectra of *Folate-4Atp-AuNP* and *Folate-MH-AuNP*.

According to this Figure, the FTIR spectra of *Folate-4Atp-AuNP* and *Folate-MH-AuNP* show the carbonyl absorbance at 1,700 cm^-1^ due to (–CONH–) and (–COO–) groups, respectively. However the –CONH– absorbance seems a bit stronger than the (–COO–) absorbance. In addition, the bands between 3000–3700 of both conjugates belong to the amine (–NH_2_) and amide (–CO–NH–) stretches of folate. Additionally, the bands below 1700 correspond to the out-of plane and in plane motions of (–NH_2_) and (**≡**C–N=) stretches of folic acid.

#### 2.1.3. Transmission Electron Microscopy

**Figure 7:**
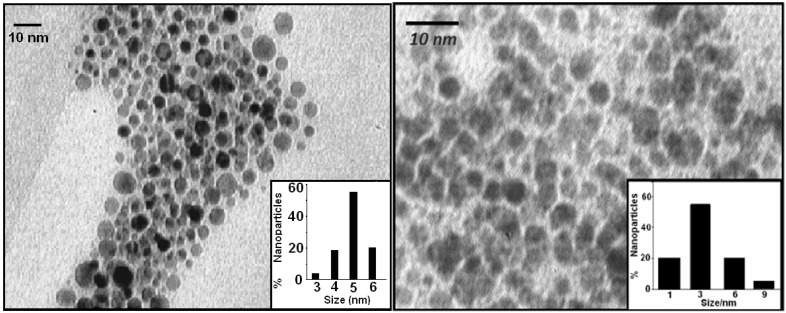
Transmission electron microscopy (TEM) photograph of Au nanoparticles in the two nanocongugates (Left: Folate-4Atp-AuNP; Right: Folate-MH-AuNP). Insets: histograms for the size distribution of the Au nanoparticles [25,26,27,28,29].

Transmission electron microscopic images of the nanoparticles were taken with a LEO 912AB instrument operated at an accelerating voltage of 120 kV with line resolution of 0.3 nm at room temperature. The samples for TEM measurements were prepared by placing a droplet of the colloidal solution onto a carbon-coated copper grid and allowing it to dry in air naturally. Based on the TEM images as shown in Figure 7, the size distributions of the final product were determined by counting at least 300 particles shown as insets in Figure 7.

According to Figure 7, the shapes of nanoparticles are quite spherical and the size histograms indicate the formation of less polydispersed nanoparticles for *Folate-4Atp-AuNP* with an average diameter of 4.8 nm than for *Folate-MH-AuNP* with an average diameter of about 3 nm.

#### 2.1.4. X-Ray Diffraction

**Figure 8:**
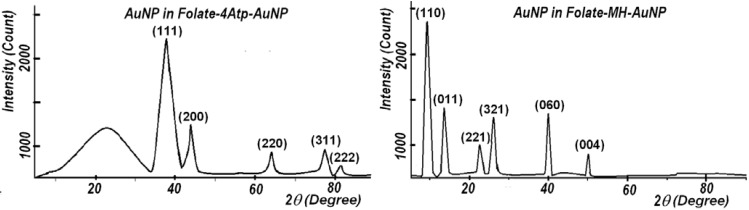
X-ray diffraction (XRD) patterns of Au nanoparticles in Folate-4Atp-AuNP (left panel) [25,26,27] and in Folate-MH-AuNP (right panel) [28,29].

X-ray diffraction was carried out with the Bruker D8 ADVANCE X-ray Diffractometer, using the wavelength of 0.15406 nm (CuKα) radiations at room temperature. As shown in Figure 8, the crystalline nature of these nanoparticles is confirmed through X-ray diffraction (XRD) analyses.

In the case of the *Folate-4Atp-AuNP* nanoconjugate, as shown in Figure 8, the Miller indices are (111), (200), (220), (311), (222) and the lattice constants are found to be a = b = c = 0.407376 nm. This structure is identified as the face-centered cubic (fcc) structure [25]. In the case of *Folate-MH-AuNP* nanoconjugate, as shown in Figure 8, the Miller indices are (110), (011), (221), (321), (060), (004) and the lattice constant is found to be a = 1.348 nm, b = 1.348 nm, and c = 0.725 nm. This structure is identified as a quasi-cubic structure.

#### 2.1.5. Elemental Analysis

The elemental analyses for carbon, hydrogen, nitrogen, sulfur and oxygen were performed using a Thermo Finnigan Flash EA CHNS-O analyzer. The gold percentages in the nanoconjugates were determined by Shimadzu model AA-670 atomic absorption spectrophotometer.

Elemental analysis of *Folate*-*4Atp*-*AuNP* and *Folate*-*MH*-*AuNP* determined by Carbon Hydrogen Nitrogen Sulfur Oxygen (*CHNS-O*) Analysis and Atomic Absorption Spectrometry resulted in the data reported in Table 1.

**Table 1:** Elemental analysis data of the nanoconjugates.

Elements→		[Au]	[C]	[H]	[N]	[S]	[O]	Total	[C]:[H]	[S]:[H]
Nanoconjugate ↓										
Folate-4Atp-AuNP	Expt’l [25,26,27]	41.3	28.7	2.5	13.3	3.5	10.7	99.9	11.48	1.4
	Stochiometric	26.5	40.3	3.1	15	4.3	10.8	100	13	1.39
Folate-MH-AuNP	Expt’l [28,29]	32	38.2	3.8	11.2	3.6	11.2	100	10.03	0.95
	Stochiometric	26.2	39.8	4	13	4.2	12.6	100	9.95	1.05

The gold molecular weight is 196.97. If one folate is conjugated to gold for the two nanoconjugates their molecular weights would be 744 for *Folate*-*4Atp*-*AuNP* and 753 for *Folate*-*MH*-*AuNP*. This will amount to 26.5% and 26.2% of gold per nanoconjugate, respectively. Accordingly, the elemental analysis of the two nanoconjugates indicated that both have one folate conjugated to each gold particle. The final powder product containing *Folate*-*4Atp*-*AuNP* has more non-conjugated gold in it than the powder containing *Folate*-*4Atp*-*AuNP*. According to this table, the non-conjugated gold content of *Folate*-*4Atp*-*AuNP* is 9.3% higher than the non-conjugated gold content of *Folate*-*4Atp*-*AuNP*. The non-conjugated AuNPs and other chemicals were removed from the systems before the *in vitro* tests reported in the next section.

#### 2.1.6. Stability Comparison

The chemical stability of the two nanoconjugates can be compared through the differences in their bond energies. The only difference in the bonds in formation of the two nanoconjugates are the N–C bond of (–NH–CO–) between 4-aminothiophenol and folic acid and the O–C bond of (–O–CO–) between 6-mercapto-1-hexanol and folic acid. The standard bond dissociation energy of the N-C bond is 73 kcal/mole and that of the O–C bond is 85.5 kcal/mole [34]. This data suggests that the *Folate-MH-AuNP* should be more stable than *Folate-4Atp-AuNP.*

### 2.2. In Vitro Tests of Nanoconjugates on Cancer Cells

The two nanoconjugates reported above were used to selectively target the folate-receptors that are overexpressed on the surface of tumor cells. In this section, we report the results of our comparative study utilizing the two nanoconjugates for the improvement of cellular internalization of *AuNP*. For this purpose, human adenocarcinoma HeLa cells were chosen as our model cancer cell line because it is known to overexpress folate-receptors [35]. HeLa cells are an immortal cell line used in medical research, which was derived from cervical cancer cells. In addition, MCF7 cell line was selected as the control because of its very low level of folate-receptor expression [36]. Actually, in this research, preferential targeting to cancerous cells by *the two nanoconjugates* was studied by comparing the results obtained from HeLa and MCF7 cell lines.

#### 2.2.1. Nanoparticle Cytotoxicity

**Figure 9:**
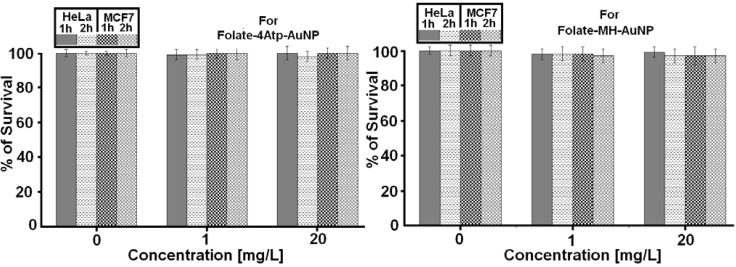
The percentage survival of HeLa and MCF7 cells incubated with different concentrations of nanoconjugates (Left: Folate-4Atp-AuNP and right: Folate-MH-AuNP) for 1 or 2 h [25,26,27,28,29].

We investigated the cytotoxicity of the two nanoconjugates on HeLa and MCF7 cell lines at various concentrations ranging from 1 to 100 μg/mL and for different incubation periods of 1, 2 and 4 hours. No significant cytotoxicity was observed in any sample even at the higher nanoparticle concentrations (100 μg/mL) for HeLa and MCF7 cells and for 1–4 hours as we reported previously [26,27,28,29,30]. In Figure 9, we report the comparative results for the two nanoconjugates for concentrations up to (20 μg/mL) and for 1 and 2 h incubation periods.

Overall, no significant cytotoxicity difference was observed between HeLa and MCF7 cell lines, as is evident from Figure 9.

#### 2.2.2. Effects of Intense Pulsed Light (IPL) Exposure to Cells

**Figure 10:**
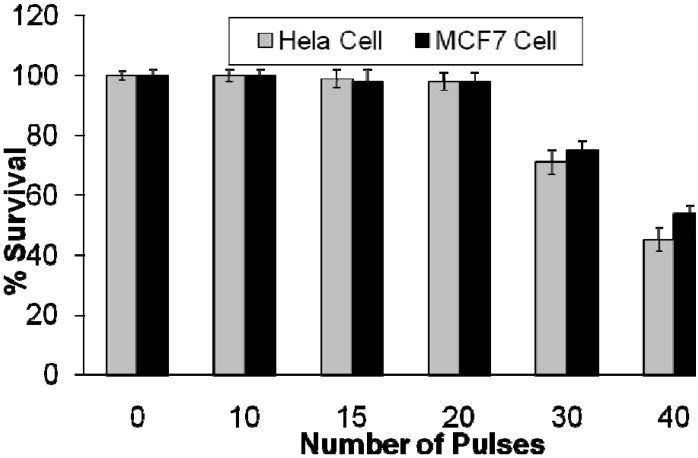
The percentage survival of HeLa and MCF7 cell lines following exposure to different number of IPL pulses with the following properties: Operating mode: single; Spot size: 8 mm × 15 mm; Fluency: 15 J/cm^2^; Filter (wavelength): 560 nm; Pulse duration: 3 milliseconds [25,26].

To examine the effect of IPL on HeLa and MCF7 cell lines, different pulse numbers were applied to both cell lines without the presence of the nanoconjugate. These examinations were conducted to observe how the number of pulses affects cell survival. For this purpose, the treatment parameters (energy fluency: 15 J/cm^2^, cut-off filter: 560 nm, pulse duration: 3 milliseconds) of each IPL pulse were selected based on preliminary studies. To test the effects of IPL exposure to cells, different pulse numbers (10, 15, 20, 30, and 40 pulses) were applied to both, HeLa and MCF7 cell lines. Figure 10 shows the results of our *in vitro* studies on HeLa and MCF7 cell lines using IPL probe without the presence of nanoconjugates.

According to Figure 10, no significant cell lethality occurred when the number of pulses, with the specified properties, was increased up to 20 pulses. The HeLa cell viability dropped from (98 ± 2)% for 20 pulses to (71 ± 4)% for 30 pulses and to (45 ± 4)% for 40 pulses. The MCF7 cell viability dropped from (98 ± 2)% for 20 pulses to (75 ± 3)% for 30 pulses and to (54 ± 4)% for 40 pulses.

Based on the data presented in Figure 10, it can be concluded that IPL exposure to both cell lines is harmless when up to 20 pulses are applied with the specifications described. As a result, we conducted our later experiments using IPL with the same properties as reported in Figure 10, *i.e.,* Operating mode: single; Spot size: 8 mm × 15 mm; Energy fluency: 15 J/cm^2^; Filter (wavelength): 560 nm; Pulse duration: 3 milliseconds; Number of pulses: 20.

#### 2.2.3. Photothermal Studies

As noted above, the *AuNP*s in the presence of appropriate light irradiation produce high temperatures. The level of temperature produced by *AuNP*s impregnated inside cancerous cells can be controlled by the level and duration of radiation in order to photothermally kill the cells. We have investigated the potential of the two nanoconjugates, which we have produced for photothermal treatment using an IPL probe. We have also investigated the differences between using the nanoconjugates along with IPL exposure for the HeLa cell line, which is folate-receptor overexpressing and the MCF7 cell line, which expresses a low level of folate-receptors.

**Figure 11:**
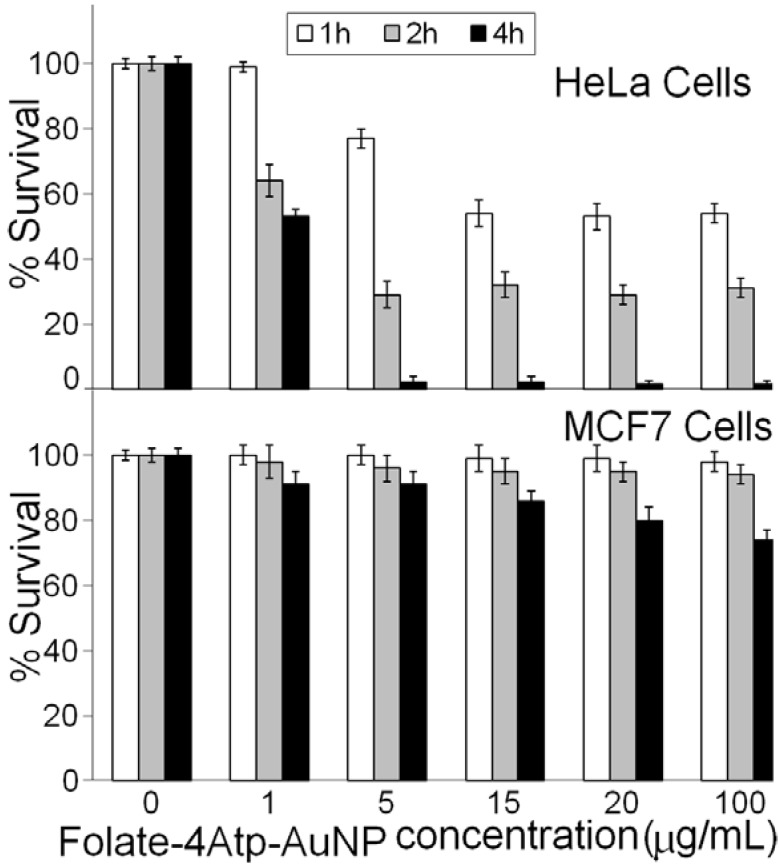
The percent survival of HeLa and MCF7 cells following photothermal treatment *versus* the Folate-4Atp-AuNP nonoconjugate concentrations and with 1, 2 and 4 h incubation periods [25,26].

In all of our *in vitro* experiments, the IPL pulse duration was kept at 3 milliseconds. We investigated the role of two factors on photothermal treatment of cells: 1. the effect of the dosage of nanoconjugates on photothermal treatment; 2. the effect of incubation period of cells with nanoconjugates. In Figure 11, we report the percentages of HeLa cells surviving following incubation with 0, 1, 5, 15, 20 and 100 µg/mL of the *Folate*-*4Atp*-*AuNP* nonoconjugate for 1, 2 and 4 hours.

According to Figure 11, for the same incubation period the percentage of live HeLa cells is dependent on the dosage of the nanoconjugates up to a dosage of 5 µg/mL. No significant difference is observed when the dosage of *Folate*-*4Atp*-*AuNP* nanoconjugate is above 5 µg/mL regardless of the Folate-4Atp-AuNP nanoconjugate concentration and the incubation period. The most significant cell death at a minimum of nanoconjugate concentration occurred after 4 h incubation and at 5 µg/mL nanoconjugate concentration. Specifically, after 4 h of incubation, the viability of HeLa cells decreased from ~55% at 1 µg/mL nanoconjugate concentration to ~1% at 5 µg/mL nanoconjugate concentration.

It should also be noted that with one hour of incubation, the nanoconjugate concentration played a role in inducing cell death up to nanoconjugate concentrations of 15 µg/mL. Concentrations over 15 µg/mL did not seem to induce more cell lethality. The fastest decrease in cell viability was observed by using 5 µg/mL of nanoconjugate concentration and changing the incubation time from one hour (77 ± 3)% to two hours (29 ± 3)%. Generally, to reach a higher level of cell death, a longer incubation time is preferred over using higher concentrations of nanoconjugate. For example, according to Figure 11, using 1 µg/mL of nanoconjugate incubated with HeLa cells for four hours and 15 µg/mL of nanoconjugate incubated with HeLa cells for one hour induce a similar cell death.

To compare the amount of cell lethality observed for HeLa cells with MCF7 cells, additional experiments were conducted. This step of the research was designed to investigate whether nanoconjugates would operate selectively for cells overexpressing the folate-receptor. Figure 11 also shows percent survival of MCF7 cells incubated with 1, 5, 15, 20, 100 µg/mL of nanoconjugate for different incubation times. All the conditions for experiments on MCF7 cells were identical to the conditions for experiments on HeLa cells. According to Figure 11, after one hour of incubation, no significant cell death was observed for MCF7 cells even at very high concentrations (100 µg/mL) of nanoconjugate. The percentage of MCF7 cells survival did not significantly alter by increasing the incubation time up to two hours. Somewhat more cell death was observed at all concentrations after four hours incubation. When the concentration increased from 1–100 µg/mL, MCF7 cell lethality varied at most from 9% (at 1 µg/mL nanoconjugate concentration) to 26% (at 100 µg/mL nanoconjugate concentration).

It is well-known that the level of folate-receptor expression and activity plays an effective role on the amount of nanoconjugates internalized into the cell. This means that a cell with a high level of folate-receptor expression on its surface can uptake more folate conjugated materials than a cell with a low level of folate-receptor expression. HeLa cells have significantly more folate-receptors than MCF7 cells [35]. Accordingly, it is expected that at the same concentration of nanoconjugate and with the same incubation time, HeLa cells can uptake more nanoconjugates than MCF7 cells. As a result, if similar conditions of light exposure apply to both cell lines, HeLa cells lethality should be higher than MCF7 cells.

By comparing the results for HeLa and MCF7 cells in Figure 11, we recognize a significant difference in the viability of HeLa and MCF7 cell lines. For example, 5 µg/mL of nanoconjugate incubated with HeLa cell for four hours can induce cell lethality of (98 ± 2)%, whereas cell lethality of (9 ± 4)% can be achieved with the same conditions in MCF7 cells. This translates to a HeLa cell lethality of about 90% more than MCF7 cells.

**Figure 12:**
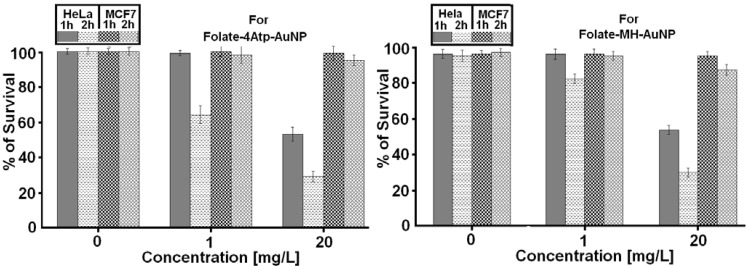
The percentage of HeLa and MCF7 cell survival following photothermal treatment *versus* Folate-4Atp-AuNP [25,26] and Folate-MH-AuNP nonoconjugates concentrations and with 1 and 2 h incubation periods.

Similar results were obtained for the *Folate-MH-AuNP* nanoconjugate. In Figure 12 we compare the results for *Folate-4Atp-AuNP* and *Folate-MH-AuNP* nanoconjugates.

According to Figure 12, *Folate-4Atp-AuNP* nanoconjugate is slightly more effective in HeLa cells than the *Folate-MH-AuNP* nanoconjugate at lower concentrations. However, at high concentrations, the two nanoconjugates behave similarly.

Based on the reported data in Figure 9, Figure 10, Figure 11, Figure 12, we conclude that the internalization (uptake) of nanoconjugates have completely taken place. Our conclusions are based on the following: (i). Figure 9 shows that when we use nanoconjugates alone and without IPL, no cytotoxicity is observed (at any concentration, at any incubation time). (ii). Figure 10 shows that when we use 20 pulses of IPL alone and without using any kind of nanoconjugates, no cytotoxicity is observed (this is true for both cell lines). (iii). From Figure 11 and Figure 12, we observe significant cytotoxicity when we use both IPL pulses and nanoconjugate together and this depends on concentration and incubation time.

## 3. Discussion

We report a comparative study of synthesis, characteristics, and *in vitro* tests of two folate-conjugated gold nanoparticles using two different linkers, *i.e.*, 4-aminothiophenol (*4Atp*) and 6-mercapto-1-hexanol (*MH*) and with two different sized AuNPs. We have previously reported on the characterization of these nanoconjugates using several spectroscopic techniques [25,26,27,28,29,30]. UV-visible and FTIR spectroscopy confirmed the attachment of folic acid to the gold nanoparticles through both of the linkers. We also confirmed the crystalline nature of the final nanoconjugate products(*Folate*-*4Atp*-*AuNP* and *Folate*-*MH*-*AuNP*) by TEM microscopy and XRD spectroscopy. After confirming folate attachment and the physical size and crystalline nature of the nanoconjugates, *in vitro* cell studies were performed.

To determine both the targeting ability and lethality of these nanoconjugates, HeLa and MCF7 cell lines were used for *in vitro* experiments. HeLa cells were chosen because they characteristically overexpress the folate-receptor. MCF7 cells were used because of their lack of folate-receptor expression. Intense Pulsed Light (IPL) technique was used to heat the nanoconjugates. The effect of IPL on the cells without the nanoconjugates was investigated to determine a number of pulses which would not cause harm to the cells. It was determined that up to 20 pulses of light would not by itself cause cell death. Therefore, for the subsequent nanoconjugate experiments, 20 pulses of light were used. The nanoparticles by themselves were not cytotoxic to either cell line after four hours incubation with 100 µg/mL of each nanoconjugate. The combination of IPL with the nanoconjugates produced maximum cell lethality after four hours incubation with the HeLa cell line while having significantly lower cell lethality in the MCF7 cells. The lowest concentration of the nanoconjugate and incubation time which caused the greatest cell death, (98 ± 2)%, in HeLa cells was 5 µg/mL at four hours. Under the same conditions, only a (9 ± 4)% lethality rate was observed in MCF7 cells. This demonstrated that the folate conjugated gold nanoparticles are selective for cells that overexpress the folate receptor. Comparing photothermal therapy with both folate-nanoconjugates showed that Folate-4Atp-AuNP outperformed Folate-MH-AuNP at low concentrations. Based on this result, Folate-4Atp-AuNP should be developed further for *in vitro* and *in vivo* work.

## 4. Future Work

Further investigation into the use of folate conjugated gold nanoparticles as a method for selectively targeting and killing cancer cells is necessary. First, imaging techniques can be utilized to show the presence or absence of the folate-nanaoconjugates within the cancer cells. This information will serve as conclusive evidence as to the fate of the nanoparticle upon entering the cell, where the particles may localize within the cell, and intracellular concentration of nanoconjugates with time. Second, processes to make the nanoconjugate more suitable for *in vivo* use must be refined. Methods may include increasing the nanoparticle size to take advantage of the Enhanced Permeability and Retention (EPR) effect of tumors and limiting free diffusion into healthy tissues. Also, PEGylation of the nanoconjugate may prevent recognition by the reticuloendothelial system and increase circulation time [21]. Third, methods of delivering the intense pulsed light to the tumors must be established. One suggestion is the use of a nanorod or nanoshell shaped gold particle due to excitation in the near infrared wavelengths (700–1100 nm) for deep non-invasive light penetration [21,22]. Another method of light delivery is through a series of optical fibers [37]. These topics must be solved before *in vivo* testing of the folate conjugated gold nanoparticles.

## 5. Conclusions

The ability of folate conjugated nanoparticles to selectively target and kill cancer cells via phototherapy is confirmed via cell based lethality experiments. The folate-nanoconjugates themselves did not induce cell death. Likewise, 20 pulses of the IPL did not cause significant cell death. Only when IPL and the nanoconjugates were both used in unison was significant cell death observed in only cells expressing the folate receptor. It was also demonstrated that folate linked to the gold nanoparticle using 4-aminothiolphenol was more effective in inducing cell death at lower concentrations than the conjugate using 6-mercapto-1-hexanol. Therefore, 4Atp as a linker should be exclusively explored further for development.
